# Supplementary material for: Integrated Bioinformatics Analysis of the Hub Genes Involved in Irinotecan Resistance in Colorectal Cancer
Source: Biomedicines. 2022 Jul 16;10(7):1720. doi: 10.3390/biomedicines10071720 (PMC9312838; doi:10.3390/biomedicines10071720)
Supplement: Supplementary file 1 [file biomedicines-10-01720-s001.zip › biomedicines-1756068-supplementary.pdf]

biomedicines

MDPI

Supplementary files:

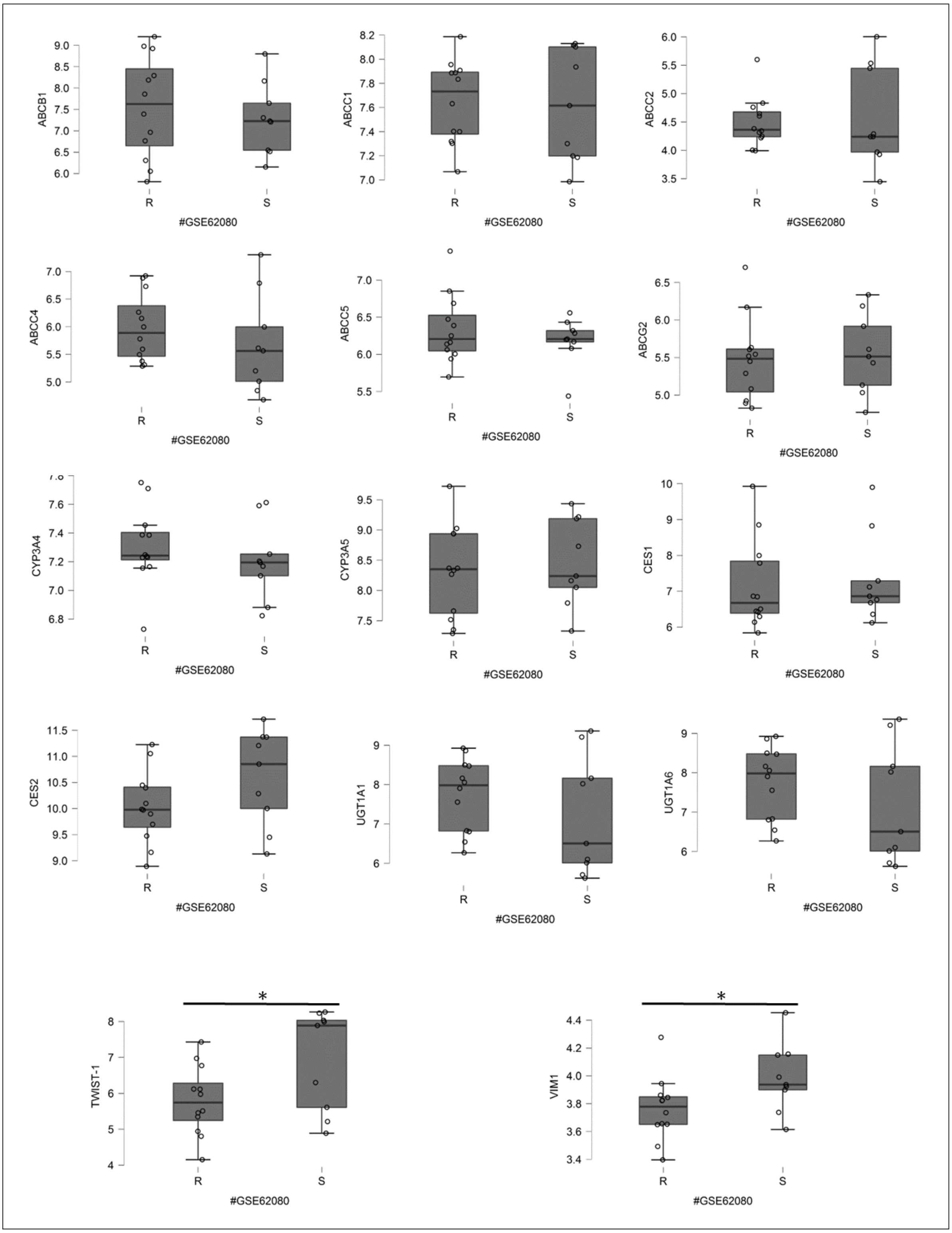

Figure S1. mRNA expression level of ABC proteins, CYP3A, CES and UGT1 variants, and EMT markers in patients treated with FOLFIRI regime. Data obtained from GEO database GSE62080. Patients treated with FOLFIRI were defined as the responders/sensitive S) and non-responders/ resistant R). Normality test (Shapiro-Wilk's) followed by Mann-Whitney U test (for not normally distributed data) or T-test (for normally distributed data). * $p > 0.05$, ** $p > 0.005$, *** $p > 0.001$.
